# Lifestyle risk factors of self-reported fibromyalgia in the Norwegian Women and Cancer (NOWAC) study

**DOI:** 10.1186/s12889-023-16773-7

**Published:** 2023-10-11

**Authors:** Faith Owunari Benebo, Marko Lukic, Monika Dybdahl Jakobsen, Tonje Bjørndal Braaten

**Affiliations:** 1 Department of Community Medicine, UiT The Artic University of Norway, Tromsø, Norway; 2 Department of Health and Care Sciences, UiT The Arctic University of Norway, Tromsø, Norway

**Keywords:** Fibromyalgia, Lifestyle, Self-report, Female, Body mass index, Overweight, Physical activity, Smoking, Alcohol consumption

## Abstract

**Background:**

While the aetiology of fibromyalgia syndrome (FM) remains unknown, lifestyle factors have been linked to the disorder. However, there are few studies on the association between lifestyle factors and FM, thus we examine the risk of self-reported fibromyalgia given selected lifestyle factors.

**Methods:**

We used data from 75,485 participants in the Norwegian Women and Cancer study. Information on FM and the lifestyle factors body mass index (BMI), physical activity level, smoking status/intensity, and alcohol consumption were obtained from baseline and follow-up questionnaires. We used Cox proportional hazards model to calculate hazard ratios (HRs) with 95% confidence intervals (CIs).

**Results:**

After a median follow-up time of 10 years, we observed 2,248 cases of self-reported fibromyalgia. Overweight (BMI 25-29.9 kg/m^2^) and obese (BMI ≥ 30 kg/m^2^) women had a relative risk of 1.34 (95% CI 1.21–1.47) and 1.62 (95% CI 1.41–1.87), respectively, compared to women with normal weight (BMI 18.5–24.9 kg/m^2^). Very low physical activity level (1–2) was associated with a 31% higher risk of self-reported fibromyalgia (HR 1.31, 95% CI 1.09–1.57) when compared to moderate physical activity level (5–6). There was a strong dose-response relationship between smoking status/intensity and self-reported fibromyalgia (p for trend < 0.001). Compared with moderate alcohol consumption (4.0–10 g/day), the risk of self-reported FM was 72% (HR 1.72; 95% CI 1.45–2.03) higher among teetotallers, and 38% (HR 1.38, 95% CI 1.23–1.54) higher among those with low consumption (0.1–3.9 g/day).

**Conclusions:**

Overweight and obesity, very low physical activity level, smoking, and alcohol consumption were associated with an increased risk of self-reported FM.

**Supplementary Information:**

The online version contains supplementary material available at 10.1186/s12889-023-16773-7.

## Introduction

Fibromyalgia syndrome (FM) is a chronic condition, characterised by the cardinal features of chronic widespread pain in at least four of five body regions, sleep disturbances, and fatigue. Other common features include cognitive dysfunction, psychiatric symptoms, somatic symptoms, autonomic regional pain syndromes, and autonomic disturbances [1]. In order to receive a diagnosis of FM, symptoms should be present at a similar level for at least 3 months, and not better accounted for by another diagnosis [1, 2]. Since its recognition as a rheumatic disease by the World Health Organisation in 1992, the definition and diagnostic criteria for FM have been revised repeatedly [2–5]. The aetiology of FM is not known, but research suggests that the main mechanism is central sensitivity to pain and reduced conditioned pain modulation [6–8], with recent indications of an autoimmune component [9]. The pain of FM is not directly attributable to a nociceptive process in the affected body regions, but it has features consistent with nociplastic pain [2, 10].

FM affects between 0.2% and 6.6% of the population worldwide, and between 2.4% and 6.8% of women [11]. It is considered the second most common musculoskeletal disorder, after osteoarthritis, for rheumatologist referral [12]. FM is more prevalent among women and tends to be higher in mid-adulthood between 30 and 50 years or after 50 years of age [13]. In Norway, studies have reported a FM prevalence of about 2.7% among men and 6.3% among women [14]. Prospective studies have also reported cumulative incidence estimates between 2.4% [15] and 3.3% [16]. FM tends to occur with comorbidities such as irritable bowel syndrome, rheumatoid arthritis, systemic lupus erythematosus, Sjogren syndrome, ankylosing spondylitis, osteoporosis, migraine, chronic fatigue syndrome, depression, and temporomandibular joint dysfunction [17, 18]. Some studies have shown a bidirectional association between FM and these comorbidities [17], while others have demonstrated a higher incidence and/or prevalence of FM in cases where these disorders are pre-existing [19, 20].

FM negatively impacts the sufferers’ quality of life. It also leads to work absenteeism, loss of productivity at home and work, disability, and even unemployment [21, 22]. Indeed, chronic pain, a hallmark of FM, affects every other dimension of health [23]. In addition, the direct and indirect cost implications of FM can be enormous on both an individual and a societal level [22, 24]. In Norway, musculoskeletal disorders are a common cause for sick leave, disability retirement, and primary care visits [14, 25, 26].

As it has for many other chronic diseases, the literature paints a multifactorial picture for FM; lifestyle, socioeconomic, and psychosocial factors have been reported to interact with biological influences and contribute to the development, and/or trajectory of symptoms [17, 27, 28]. Lifestyle factors, which are the focus of this study, have been linked to various musculoskeletal disorders [29–31]. In Norway, results from the Nord-Trondelag Health Study (HUNT Study) have shown that high body mass index (BMI) and low physical activity level significantly increase the risk of self-reported FM [15, 16]. Other studies have identified high BMI, smoking, and alcohol abstinence as risk factors for FM [17, 32].

There is a growing interest in understanding the role of lifestyle factors in the development of FM. Previous studies have provided limited information on the potential influence of lifestyle factors on FM risk. Some studies utilized a case-cohort methodology in hospital-based populations, with specific patient cohorts. Other researchers have employed prospective study design, but they only utilise baseline measurement of the studied lifestyle factors [17]. Thus, the present study is novel in that we aimed to examine the risk of self-reported FM in the presence of selected lifestyle factors in a population-based prospective cohort of Norwegian adult women, utilizing repeated measures of the lifestyle factors to account for changes over time. The findings of this study can provide important insights and contribute to the growing knowledge in the research on FM, highlighting the importance of lifestyle modifications.

## Methods

### Study sample

The Norwegian Women and Cancer (NOWAC) study is a population-based prospective cohort study that recruited more than 172,000 women between 1991 and 2007 (overall response rate: 52.7%). Women aged 30–70 years were randomly selected from the Norwegian Population Register and sent an invitation to participate, along with information about the study, and a questionnaire of between two and eight pages. Those who consented and returned a completed questionnaire were enrolled in the NOWAC study, and they were sent follow-up questionnaires approximately every 6th year thereafter. A subgroup of women received a fourth questionnaire in 2017, 12 years after the third one. Thus, depending on their time of enrolment, NOWAC participants have completed between one and four questionnaires. The study design, materials, and procedures, including validation studies, have been described in detail elsewhere [33, 34]. The Regional Committee for Medical Research Ethics and the Norwegian Data Inspectorate approved the NOWAC study.

**Fig. 1:**
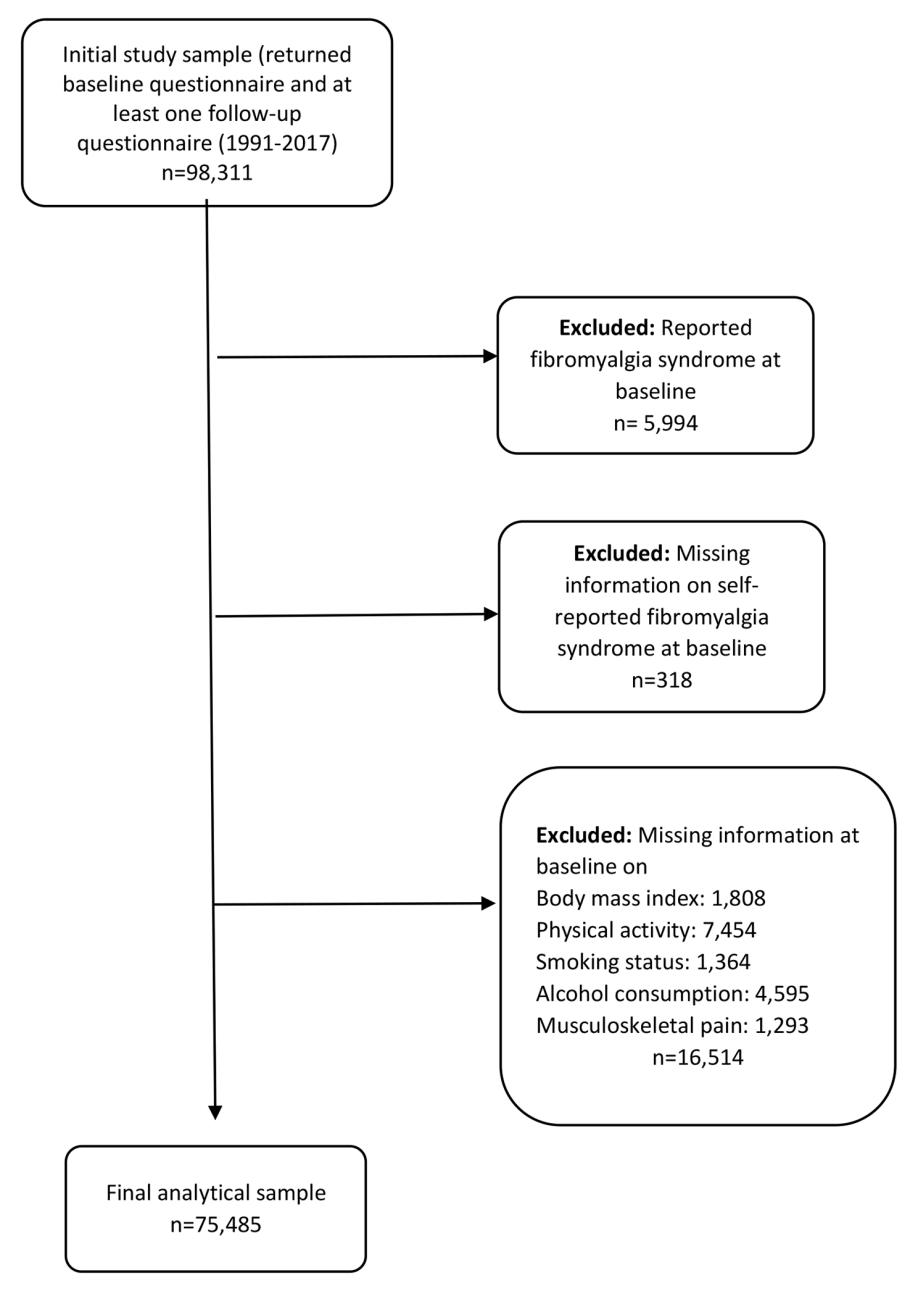
Flowchart of study participants

For the present analysis, we selected women who completed the enrolment (baseline) questionnaire in 1991–1992, 1996–1997, or 2003–2004, and at least one follow-up questionnaire. A total of 98,311 women met these criteria and comprised the initial study sample. We then excluded women with self-reported FM at baseline (n = 5,994), as well as those with missing information on self-reported FM (n = 318), BMI (n = 1,808), physical activity level (n = 7,454), smoking status/intensity (n = 1,364), alcohol consumption (n = 4,595), and musculoskeletal pain (n = 1,293) at baseline. Thus, the final analytical sample consisted of 75,485 women (Fig. 1).

### Outcome variable

Baseline questionnaires included the question, *“Have you had any of the following illnesses: fibromyalgia … (among other conditions)?”* Respondents who answered “yes”, were excluded from the study, only those who answered “no” were included in the study. The main outcome variable was self-reported FM at follow-up. The follow-up questionnaires included the instruction, *“For the following conditions: Fibromyalgia … (among other conditions), tick which year they emerged.”* Participants who ticked the year of occurrence were categorised as reporting FM, while those who did not tick any year were categorised as not reporting FM.

### Exposure variables

We selected potential lifestyle factors associated with self-reported FM as exposure variables, based on findings from previous studies [17]. They included BMI, physical activity (PA) level, smoking status/intensity, and alcohol consumption. Information on these factors was extracted from baseline and follow-up questionnaires.

We calculated BMI by dividing self-reported weight in kg by self-reported height in m^2^ and separated the variable into four categories: underweight (< 18.5 kg/m^2^), normal weight (18.5–24.9 kg/m^2^), overweight (25-29.9 kg/m^2^), and obese (≥ 30 kg/m^2^). This categorisation is recommended by the World Health Organisation [35]. Normal weight was used as the reference group. Self-reported weight and height in the NOWAC cohort have been validated previously [36].

PA level was measured on a 10-point ordinal scale via the following instruction: *“Please indicate the level of your physical activity on a scale from very low to very high by age 14, 30 and today. The scale goes from 1–10. By physical activity we mean both work in and outside the home, as well as training/exercise and other physical activity, such as walking, etc. Please mark the number that best describes your level of physical activity; 1 being very low and 10 being very high.”* We categorised PA level as very low [1, 2], low [3, 4], moderate [5, 6], high [7, 8], and very high [9, 10]. Moderate PA level was used as the reference group. This PA scale measures the total amount of PA across different domains including recreation, occupation, transportation, and household in one global score. A validity study showed a moderate, but significant Spearman’s rank correlation (range: 0.36–0.46; p < 0.001) between the scale used in the present study and objective measures of PA [37].

Smoking status was assessed by the question, *“Have you ever smoked?”* Participants who responded yes were categorised as ever smokers, and those who responded no were categorised as never smokers. Other information on current and past smoking status, and pack-years of smoking (i.e., intensity: the number of cigarettes smoked per day divided by 20 and multiplied by years of smoking) was also extracted for ever smokers. For the analyses, participants were categorised by combining smoking status and intensity: never smokers, former smokers < 10 pack-years, former smokers ≥ 10 pack-years, current smokers < 10 pack-years, and current smokers ≥ 10 pack-years. Never smokers were designated as the reference group.

Alcohol consumption was assessed by the question, *“Are you a teetotaller?”* (Teetotaller means one who never drinks alcohol/ completely abstains from alcohol consumption). For those who reported they were not teetotallers, alcohol consumption in g/day was computed from reported intake of different beverages in predefined categories. These variables were then combined and divided into the following consumption categories: teetotaller; low (0.1–3.9 g/day); moderate (4.0–10 g/day); and high (> 10 g/day). Teetotallers were used as the reference group. The limit for daily alcohol consumption was set at 10 g/d for women based on the Norwegian guidelines on Diet, Nutrition and Physical activity [38].

### Covariates

We adjusted for depression (yes, no), history of musculoskeletal pain (yes, no) and marital status (married/cohabiting, unmarried). Studies have shown an association between depression and lifestyle factors [39, 40]. Moreover, depression is frequently associated with FM; in some cases, it is considered a symptom of FM, while in others it is a comorbidity [17, 41]. Prior musculoskeletal pain (another site, acute or chronic) is thought to be the most important clinical risk factor for the development of chronic pain, and chronic pain is a cardinal feature of FM [1, 23, 42].

### Statistical analyses

We describe baseline characteristics in the final analytical sample according to self-reported FM at follow-up. Categorical variables are presented as frequencies with percentages, and continuous variables as means with standard deviations or median with interquartile range. We used Cox proportional hazards models to estimate the hazard ratios (HRs) for the association between the selected exposure variables and self-reported FM at follow-up. We interpreted relative hazards as estimates of risk ratios, with their corresponding 95% confidence intervals (CIs). We used attained age as the timescale, and participants were followed from baseline until self-reported onset of FM or the last follow-up, whichever came first.

Baseline and all repeated measures of lifestyle factors data were utilised in data analyses, to account for changes in the selected exposure variables. Baseline information was used until follow-up information became available, and follow-up information was used until self-reported onset of FM or the last follow-up, whichever occurred first [43]. We performed univariable analyses for each exposure variable, followed by multivariable analyses, mutually adjusting for the exposure variables and for depression. At each follow-up, we used lifestyle factors measured prior to that follow-up. To test for trend, we modelled the exposure variables with the median value in each group as the group indicator, except for PA level, which was modelled as a continuous variable. The proportional hazards assumption of the Cox proportional hazards models was tested using the Schoenfeld residuals tests, which showed that the covariate, history of musculoskeletal pain (p < 0.001), did not satisfy the proportional hazards assumption. Therefore, we did not adjust for this covariate in the model; instead, we stratified our final model by history of musculoskeletal pain.

Given that this is a prospective study, we assumed that missing data were not related to the outcome, and that complete-case analyses would suffice for the final multivariable model. However, we performed sensitivity analyses by conducting chained multiple imputation for missing exposure variables and repeated the analyses based on 20 imputed datasets. We also recalculated the HRs after excluding cases of self-reported FM that occurred within the first two years of follow-up (234 cases), to assess the influence of pre-existing disease on the estimated associations. Furthermore, the diagnostic criteria of FM have evolved over time, with revision of the guidelines a couple of times between 1990 and 2019 [44]. We restricted the analyses to data collected between 1991 and 2011. The 1990 diagnostic criteria were in effect and up to 85% of the FM cases were reported within this period. We lacked data on comorbidities for all participants at baseline, thus we repeated our analyses on a subsample (n = 32,959) of participants whose baseline questionnaire included questions on some comorbidities (rheumatoid arthritis, Crohn’s disease, ulcerative colitis and psoriasis). Participants were categorised as no comorbidity, one comorbidity and two or more comorbidities. All p-values were two tailed, and p < 0.05 was considered significant. All statistical analyses were performed with in STATA version 16.0 (Stata Corp, College Station, TX, USA) [45].

## Results

Baseline prevalence in the initial study sample was 6.1% (5,994 out of 98,311 women). During 979,463 person-years of observation in the final analytical sample, 2,248 women reported FM at follow-up (overall incidence rate: 229.5 per 100,000 person-years; cumulative incidence 3.0%), and the median time to event was 5(IQR = 5) years. Mean age at enrolment was 46.5 (± 8.3) years, and the median follow up time was 10(IQR = 13) years.

After adjusting for age, women with self-reported FM were more often overweight and obese compared with those who did not report FM. A larger proportion of women who reported FM were current smokers, had low or no alcohol intake, and very low, low, or very high PA levels at baseline (Table 1).

**Table 1:** Crude and age-adjusted selected baseline characteristics of the final analytical sample (N = 75,485) among women who did and did not report fibromyalgia syndrome (FM) at follow-up. The Norwegian Women and Cancer study

Characteristics	Crude		Age-adjusted proportions	
	Self-reported FM (n = 2,248) N (%)	No self-reported FM (n = 75,237) N (%)	Self-reported FM (n = 2,248) %	No self-reported FM (n = 75,237) %
**Age at enrolment (years)**				
30–39 40–49 50–59 ≥ 60	794 (35.3) 1,187 (52.8) 242 (10.8) 25 (1.1)	16,870 (23.0) 32,497 (44.4) 17,930 (24.5) 5,490 (8.1)	35.3 52.8 10.8 1.1	23.0 44.4 24.5 8.1
**Body mass index (kg/m** ^**2**^ **)**				
Underweight (< 18.5) Normal weight (18.5–24.9) Overweight (25.0-29.9) Obese (≥ 30.0)	49 (2.2) 1,474 (65.6) 540 (24.0) 185 (8.2)	1,723 (2.4) 50,327 (68.7) 16,781 (22.9) 4,406 (6.0)	1.6 61.4 27.3 9.4	2.1 69.5 21.9 5.6
Body mass index (kg/m^2^), mean (± SD)	24.0 (± 3.9)	23.7 (± 3.7)	24.5 (3.5)	23.7 (± 3.5)
**Physical activity level**				
Very low (1–2) Low (3–4) Moderate (5–6) High (7–8) Very high (9–10)	152 (6.8) 498 (22.2) 934 (41.6) 520 (23.1) 144 (6.4)	3,226 (4.4) 14,887 (20.3) 31,052 (42.4) 19,395 (26.5) 4,677 (6.4)	7.0 22.6 41.4 22.9 6.2	4.4 20.3 42.4 26.5 6.4
**Smoking status/intensity** ^a^				
Never Former < 10 pack-years Former ≥ 10 pack-years Current < 10 pack-years Current ≥ 10 pack-years	664 (29.5) 579 (25.8) 114 (5.1) 410 (18.2) 481 (21.4)	27,327 (37.3) 19,589 (26.8) 4,296 (5.9) 10,238 (14.0) 11,787 (16.1)	30.9 25.6 5.9 14.0 21.8	37.2 26.8 5.3 12.7 16.1
Number of pack-years, median (IQR)	4(11)	2(9)		
**Alcohol consumption (g/day)**				
Teetotaller Low (0.1–3.9) Moderate (4.0–10) High (> 10)	228 (10.1) 1,508 (67.1) 355 (15.8) 157 (7.0)	6,889 (9.4) 44,765 (61.1) 15,881 (21.7) 5,702 (7.8)	11.0 66.0 16.0 7.0	9.2 61.2 21.7 7.8
Alcohol consumption (g/day), median (IQR)	1.4 (3.3)	1.9 (4.5)		
**Depression**				
No Yes	1,547 (68.8) 701 (31.2)	62,028 (84.7) 11,209 (15.3)	69.7 30.3	84.7 15.3
**History of musculoskeletal pain**				
No Yes	1,431 (63.7) 817 (36.3)	63,749 (87.0) 9,488 (13.0)	61.8 38.2	87.2 12.8

^a^Smoking intensity: the number of cigarettes smoked per day divided by 20 and multiplied by years of smoking (pack-years). SD: standard deviation, IQR: interquartile range

High BMI and smoking were positively associated with self-reported FM. After multivariable adjustment, women classified as overweight or obese had a higher risk of self-reported FM when compared to women with normal weight (HR 1.34, 95% CI 1.21–1.47 and 1.62, 95% CI 1.41–1.87, respectively: p for trend < 0.001). When compared with moderate PA level, women with a very low PA level had a 31% higher risk of self-reported FM (HR 1.31, 95% CI 1.09–1.57). The HRs for low, high, and very high PA levels were not associated with self-reported FM after multivariable adjustment.

Compared with never smokers, all other categories of smoking status/intensity showed a significantly higher risk of self-reported FM. Current smokers with ≥ 10 pack-years had a 61% (HR 1.61, 95% CI 1.43–1.81) higher risk of self-reported FM, while current smokers with < 10 pack-years had a 50% (HR 1.50, 95% CI 1.31–1.71) higher risk. Former smokers with ≥ 10 pack-years had a 36% (HR 1.36, 95% CI 1.14–1.63) higher risk of self-reported FM, while former smokers with < 10 pack-years had a 16% (HR 1.16, 95% CI 1.04–1.30) higher risk. For alcohol consumption, teetotallers had a 72% (HR 1.72, 95% CI 1.45–2.03) higher risk of FM, while those who reported low alcohol consumption had a 38% (HR 1.38, 95% CI 1.23–1.54) higher risk compared to moderate alcohol consumption. High alcohol consumption (> 10 g/day) was not associated with self-reported FM (Table 2).

**Table 2:** Age-adjusted and multivariable-adjusted relative risk of self-reported fibromyalgia syndrome at follow-up according to selected lifestyle factors. The Norwegian Women and Cancer study

Variable	Age-adjusted	Multivariable^b^	P for trend
	HR (95% C1)	HR (95% C1)	
**Body mass index (kg/m**^**2**^)			
Underweight (< 18.5) Normal weight (18.5–24.9) Overweight (25.0–29.9) Obese (≥ 30.0)	0.84 (0.60–1.17) 1 (ref.) 1.43 (1.30–1.58) 1.99 (1.73–2.29)	0.75 (0.54–1.05) 1 (ref.) 1.34 (1.21–1.47) 1.62 (1.41–1.87)	< 0.001
**Physical activity level**			
Very low (1–2) Low (3–4) Moderate (5–6) High (7–8) Very high (9–10)	1.74 (1.45–2.08) 1.21 (1.08–1.35) 1 (ref.) 0.92 (0.83–1.02) 1.11 (0.93–1.33)	1.31 (1.09–1.57) 1.08 (0.97–1.20) 1 (ref.) 0.99 (0.89–1.10) 1.15 (0.96–1.37)	0.04
**Smoking status/intensity** ^a^			
Never Former < 10 pack-years Former ≥ 10 pack-years Current < 10 pack-years Current ≥ 10 pack-years	1 (ref.) 1.15 (1.03–1.28) 1.43 (1.20–1.70) 1.46 (1.28–1.67) 1.70 (1.52–1.91)	1 (ref.) 1.16 (1.04–1.30) 1.36 (1.14–1.63) 1.50 (1.31–1.71) 1.61 (1.43–1.81)	< 0.001
**Alcohol consumption (g/day)**			
Teetotaller Low (0.1–3.9) Moderate (4.0–10) High (> 10)	1.72 (1.46–2.03) 1.47 (1.32–1.64) 1 (ref.) 1.08 (0.90–1.29)	1.72 (1.45–2.03) 1.38 (1.23–1.54) 1 (ref.) 1.00 (0.84–1.20)	< 0.001

^a^Smoking intensity: the number of cigarettes smoked per day divided by 20 and multiplied by years of smoking (pack-years). ^b^Multivariable model adjusted for marital status and depression at baseline. HR: hazard ratio, CI: confidence interval

Exclusion of participants who reported FM within the first two years of follow-up (n = 243) did not appreciably change the risk estimates (see Supplementary Tables 1, Additional file 1) for the entire analytical sample; thus, these participants were retained in the final analyses. Furthermore, results from analyses with the imputed datasets and the complete-case analyses were similar. Results from analyses accounting for the diagnostic period were comparable to those from the entire dataset. Findings from analyses on the subsample (n = 32,959) that had information on comorbidities at baseline, were similar to results of analyses with the whole analytical sample (see Supplementary Tables 2, Additional file 1).

## Discussion

The present report analysed data from the NOWAC study to examine the association between self-reported FM and the lifestyle factors BMI, PA, smoking status/intensity, and alcohol consumption. Using repeated measures, our results showed that overweight, obesity, very low PA level, smoking, teetotaller, and low alcohol consumption were significantly associated with self-reported FM. BMI and smoking showed a positive association with self-reported FM, while PA and alcohol consumption had negative associations.

Our study revealed a baseline prevalence of self-reported FM of 6.1% in the initial study sample, an incidence rate of 229.5 per 100,000 person-years and a cumulative incidence of 3% in the final analytical sample. This is comparable to estimates from a nationally representative population-based Norwegian survey, that reported the prevalence of about 6.3% among women [14]. One Norwegian study of women aged 20–49 years reported an incidence rate of 583 per 100,000 [46]. This is clearly higher than the incidence rate we observed in the present study, perhaps since our participants were older, and the proportion of women with self-reported FM at baseline was higher, which led to their exclusion from the final analytical sample. Studies on FM from other countries have reported prevalence estimates in the general population between 0.2% and 9.3%, with a consistently higher prevalence among women [13, 17]. The wide range of prevalence and incidence estimates across studies may be due to differences in the definition of FM cases, the study setting, or how information on FM is collected.

The current study revealed that overweight and obesity were associated with self-reported FM, which is comparable to findings from previous cohort studies. A Norwegian health survey investigated the longitudinal association between obesity and FM after an average follow-up of 11 years. The results showed a significantly higher risk of FM among obese compared to normal-weight women [15]. Results from the Finnish twin cohort study demonstrated an increased risk of FM with obesity and overweight [47]. Several studies have also reported positive associations between obesity and various musculoskeletal disorders [48].

The relationship between BMI and FM is not fully understood. One possible mechanism is inflammation. Evidence suggests that inflammation plays a role in the pathogenesis of fibromyalgia [49], and obesity is a proinflammatory state that can produce hyperalgesia through adipocyte-driven systemic inflammatory activity involving proinflammatory chemical mediators. Studies have demonstrated elevated levels of such chemical mediators as IL-6, IL-8, TNF-alpha, leptin in both obese subjects and those with FM [49–52]. These substances influence multiple central and peripheral neural pathways, subsequently altering sensitivity to pain. Proinflammatory cytokines are also thought to influence fatigue, mood changes, and cognitive function, all of which are features of FM [50, 53, 54]. Dysregulation of the hypothalamic-pituitary axis (HPA) and subsequent altered stress response may also be a mechanism through which overweight/obesity may influence FM. In obesity, metabolic dysregulation and chronic inflammation may impose chronic stress on the HPA, with eventual hypoactivity of the axis, which is also observed in FM. This may act through several mechanisms to disrupt normal circadian rhythms, and thus contribute to sleep disturbances and fatigue [55, 56].

The current study also found that, compared with moderate PA level, only very low PA level, was associated with increased risk of self-reported FM. There was also there was a significant dose-response relationship (p for trend = 0.04) between PA and self-reported FM. The trend of association observed was U-shaped, suggesting that the risk of self-reported FM may be increased at extremely low or high PA levels. This U-shaped trend has also been reported in other chronic musculoskeletal pain conditions [57, 58]. Conversely, previous studies reported no association between exercise and FM symptoms, neither did they observe a dose-response relationship [15, 47]. Our study measured PA level differently, using a validated PA scale that comprised the domains of recreation, occupation, and household PA. This may explain the differences between our results and those of the cited studies.

The precise mechanism is unclear, but PA may impact the risk of FM via activation of endogenous pain inhibition pathways. Evidence in the literature suggests that during acute episodes of exercise in healthy subjects, endogenous opioids are released, which modulate pain processing areas in the brain and spinal cord. This subsequently tends to reduce sensitivity to pain [59, 60]. Moderate PA may also act through the reduction of proinflammatory cytokines to modulate pain. This may be particularly beneficial among those who are overweight/obese [61, 62]. Mork et al. reported that the risk of FM among women with overweight/obesity was attenuated by physical exercise [15]. Although physical exercise is recommended for the management of FM [63], it is not clear if PA has cumulative effects that can protect against the development of chronic musculoskeletal pain. In the literature, the relationship between PA and musculoskeletal pain conditions is often not significant. It is possible that the relationship is more complex and indirect; even mediated by other factors.

In the present study, we also found positive association between all categories of smoking status/intensity and self-reported FM, compared with never smoking, with a strong dose-response relationship. Similarly, Choi et al. found that past vs. never smoking was positively associated with incident fibromyalgia [32]. Smoking has also been reported to increase the risk of other chronic musculoskeletal pain conditions [29, 64, 65]. The relationship between smoking and FM may be mediated through the effect of nicotine on the opioid, serotoninergic, and HPA systems, which in turn influence pain, sleep, anxiety, and cognition. Short-term exposure to nicotine from cigarette smoke may produce analgesia. However, prolonged exposure may desensitise nicotine receptors, downregulate the HPA, and inhibit activation of the opioid and serotoninergic systems, subsequently increasing susceptibility to pain [66–68]. Also smoking has been associated with an imbalance in the interaction between the proinflammatory adipokine, leptin and neuropeptide Y, which may play a role in chronic pain mechanism [69].

Compared with moderate alcohol consumption (4.0–10 g/day), the risk of FM was higher among teetotallers and low alcohol consumption category, in the current study. In contrast, the prospective Adventist Health study with 25 years of follow-up conducted in the US, reported that alcohol had a positive, but non-significant, association with FM [32]. This difference in results may be because most of the subjects in the Adventist Health study were abstainers. Similar to our findings, studies have reported protective effect of weekly alcohol consumption against chronic widespread pain, lower severity of FM symptoms and better quality of life, with low and moderate alcohol consumption [70, 71]. Mundal et al. suggested that abstention from alcohol may be associated with less socialising, and subsequently poorer health [72], which may explain the association we observed in the index study. However, the apparent protective effect of moderate alcohol consumption observed by us and others may be due to the inclusion of former drinkers who quit drinking or reduced their alcohol intake, perhaps due to health problems [73]. Unfortunately, we cannot ascertain this, as we do not have data on former drinkers. The association we observed between alcohol consumption and self-reported FM may also be due to other unmeasured factors, such as comorbidities, for which we do not have adequate data. While statistical results suggest that moderate alcohol consumption may be negatively associated with FM, non-drinkers are not advised to start drinking, as alcohol consumption is associated with other negative health outcomes [74].

### Strengths and limitations

The strength of this study is the use of data from a large, nationally representative population-based sample of women, with prospective repeated measures of lifestyle factors, which allowed us to account for changes in these factors during follow-up. Since lifestyle factors most likely vary over time, the use of repeated measures makes for a more robust analysis, as it utilises all available data and accounts for variations across the follow-up period [43]. The use of a prospective design minimises differential recall of lifestyle factors between women who reported FM and those who did not. Furthermore, validation studies have been conducted for most lifestyle factors measured in the NOWAC study [36, 37].

There are some limitations in our study. Data on FM were obtained from self-report, in response to a single question. The question has not been formally validated, and there was no confirmatory diagnosis of FM in the study setting. The symptoms of FM are shared with many other musculoskeletal conditions; thus one cannot rule out that some persons may not be aware that they have it or may confuse it with other conditions [75]. Participants who reported FM, were also asked to recall the year of onset of FM. Recall bias might result in inaccurate estimate of time to event. Also, we do not know if those who responded “yes” to the FM question were diagnosed using formal diagnostic criteria. The NOWAC study was initiated in 1991 [34], and the diagnostic criteria for FM were revised a couple of times between 1991 and 2017 (i.e., the end point for this study) [3–5]. Thus, if a person was clinically diagnosed, the resultant variation in diagnostic criteria may have influenced the diagnosis of FM, based on the period in which the person was diagnosed.

Data on lifestyle factors were self-reported, so we cannot rule out the presence of measurement errors. There may be some under- or overreporting due to social desirability bias, which may have led to misclassification. However, the alcohol consumption, BMI and PA measures used in this study have been validated [76]. Furthermore, in epidemiological studies, lifestyle data are usually collected via self-reports, as it is often the most feasible method. In addition, we cannot completely rule out the possibility that FM preceded lifestyle factors. It takes an average of 2.3 years to be diagnosed with FM, thus symptoms may be present without a diagnosis, influencing lifestyle [77]. We had about 16% missing data on our selected lifestyle factors at baseline, and conducted multiple imputation, assuming that our data were missing at random. However, there is a probability that some of our data may not have been missing at random, which could introduce bias in the results from the imputed datasets.

Lastly, unmeasured, and residual confounding cannot be completely ruled out. In our analyses, we did not adjust for family history of FM, which is a significant predictor, given the evidence of familial clustering for this disorder [47]. We also had limited or no data on relevant medical comorbidities and sleep problems, which have been associated with FM [17]. Our observed association may be biased by unmeasured confounding, if the distribution of unmeasured factors is different for those that reported and those who did not report FM. Thus, the results should be interpreted with caution.

## Conclusion

In this prospective analysis of lifestyle factors and self-reported FM risk in the NOWAC study, overweight and obesity, very low PA level, smoking status/intensity, teetotaller and low alcohol consumption were associated with an increased risk of FM. Our results align with previous literature on lifestyle factors and the risk of FM, while accounting for changes in lifestyle factors over time.

From a medical perspective, our findings can equip health care providers with relevant knowledge to effectively manage those that may be at risk of FM. Discussions about these modifiable lifestyle factors can empower individuals to proactively take steps to reduce their risk. Our study also contributes to the wider context of fibromyalgia prevention. All the modifiable risk factors we identified can be addressed through targeted prevention and health promotion initiatives. For example, interventions that advocate and empower individuals to maintain a healthy weight, abstain from, and quit smoking, may contribute to decreasing the risk of FM and potentially reduce the burden of fibromyalgia on the society. Although its association with FM was not strong, physical activity is still a tool that can be used to for weight management. Further studies can examine the impact of a combination of lifestyle factors on the risk of FM.

### Electronic supplementary material

Below is the link to the electronic supplementary material.

Supplementary Material 1

## Data Availability

The data that support the findings of this study are not publicly available. Ethical and legal restrictions apply to the availability of these data, which were used under license for the current study. However, researchers can apply for access to data from NOWAC Study following guidelines provided at the website - https://uit.no/research/nowac. Enquiries about the NOWAC Study can be sent by email to the advisor - bente.isaksen@uit.no.

## List of Abbreviations

BMI: Body Mass Index
CI: Confidence Interval
FM: Fibromyalgia syndrome
HPA: Hypothalamic-pituitary axis
HR: Hazard Ratio
HUNT: Helseundersøkelsen I Trøndelag
IL-6: Interleukin − 6
IL-8: Interleukin − 8
IQR: Interquartile Range
NOWAC: Norwegian Women and Cancer
NSD: Norsk Senter for forskningdata (Norwegian Center for Research Data)
PA: Physical Activity
REK: Regional Committees for Medical and Health Research Ethics
SD: Standard Deviation
TNF-alpha: Tumor Necrosis Factor- alpha
